# A Herpes Simplex Virus 2 (HSV-2) Glycoprotein D-expressing Nonreplicating Dominant-Negative HSV-2 Virus Vaccine Is Superior to a gD2 Subunit Vaccine against HSV-2 Genital Infection in Guinea Pigs

**DOI:** 10.1371/journal.pone.0101373

**Published:** 2014-06-30

**Authors:** Pengwei Zhang, Lining Xie, John W. Balliet, Danilo R. Casimiro, Feng Yao

**Affiliations:** 1 Department of Surgery, Brigham and Women's Hospital, and Harvard Medical School, Boston, Massachusetts, United States of America; 2 Vaccine Research, Merck Research Laboratories, Merck & Co., Inc., West Point, Pennsylvania, United States of America; Geisel School of Medicine at Dartmouth, United States of America

## Abstract

We recently constructed a novel non-replicating dominant-negative HSV-2 recombinant viral vaccine (CJ2-gD2) capable of expressing various HSV-2 antigens that are dominant targets of HSV-2-specific CD8 T-cell response. Importantly, CJ2-gD2 expresses gD2, the HSV-2 major antigen glycoprotein D, as efficiently as wild-type HSV-2 infection and can lead to a nearly 500-fold reduction in wild-type HSV-2 viral replication in cells co-infected with CJ2-gD2 and wild-type HSV-2. In this report, we show that CJ2-gD2 elicits a strong antibody response to various HSV-2 antigens and is highly effective in the prevention of primary and recurrent HSV-2 genital infection and disease in the immunized guinea pigs. The direct comparison study between CJ2-gD2 and a gD2 subunit vaccine (gD2-alum/MPL) with a formulation akin to a vaccine tested in phase III clinical trials shows that CJ2-gD2 is 8 times more effective than the gD2-alum/MPL subunit vaccine in eliciting an anti-HSV-2 specific neutralizing antibody response and offers significantly superior protection against primary and recurrent HSV-2 genital infections. Importantly, no challenge wild-type HSV-2 viral DNA was detectable in dorsal root ganglia DNA isolated from CJ2-gD2-immunized guinea pigs on day 60 post-challenge. CJ2-gD2 should be an excellent HSV-2 vaccine candidate for protection against HSV-2 genital infection and disease in humans.

## Introduction

Genital herpes is one of the most common sexually transmitted diseases worldwide and is the primary cause of genital ulcer disease in both developed and developing countries [1]. Herpes simplex virus type 2 (HSV-2) is the primary cause of recurrent genital herpes. Approximately fifty to sixty million adults in the United States are infected with HSV-2 [2], [3]. HSV infections can cause significant clinical problems in AIDS and cancer patients, organ transplant recipients, and newborns [4]. Moreover, genital HSV-2 infection triples the risk for sexual acquisition and transmission of HIV infection [5]. Currently, no antiviral therapy is effective in preventing or reducing the incidence of symptomatic and asymptomatic HSV-2 recurrent infections with the exception of daily suppressive therapy. Thus, there is a strong need to develop safe and efficacious vaccines against HSV-2 infection [1], [6], [7].

Using the T-REx gene switch technology (Invitrogen Inc., CA) developed in this laboratory, we constructed a novel class of replication-defective HSV-1 recombinants capable of blocking wild-type HSV-1 and HSV-2 infections (dominant-negative) [8], [9]. CJ9-gD is a prototype of a non-replicating dominant-negative HSV-1 viral vaccine, which encodes 2 copies of the dominant-negative mutant polypeptide UL9-C535C of HSV-1 origin of viral replication binding protein UL9 under the control of the tetO-containing hCMV immediate-early promoter (CMVTO) and a single copy of the HSV-1 glycoprotein D (gD) gene under driven by CMVTO [10]. CJ9-gD is completely replication-defective, cannot establish detectable latent infection *in vivo*, and expresses high levels of HSV-1 major antigen glycoprotein D (gD) independent of HSV-1 viral DNA replication [10]. Immunization with CJ9-gD elicits a strong and long-term protective immune response against HSV-1 infection in mouse and guinea pig models of HSV-1 infections [10]–[12].

Given these demonstrated favorable safety and immunological profiles of CJ9-gD and the hypothesis that HSV-2 recombinant virus-based vaccine would be significantly superior to the HSV-1 counterpart in protecting against HSV-2 infection, we recently constructed an HSV-2-based non-replicating dominant-negative recombinant virus, CJ2-gD2. To maximize the HSV-2 glycoprotein D (gD2) expression by CJ2-gD2, we replaced both copies of the HSV-2 ICP0 gene with a bi-directional transcription unit that encodes the full-length gD2 gene driven by the tetO-bearing HSV-1 ICP4 promoter and the UL9-C535C gene under the control of CMVTO [13]. While CJ2-gD2 expresses little gD2 in tetracycline repressor (tetR)-expressing cells, it expresses gD2 as efficiently as wild-type HSV-2 infection in non tetR-expressing cells. CJ2-gD2 is avirulent and incapable of establishing detectable latent infection following immunization. We demonstrate that high-level expression of gD2 by CJ2-gD2 leads to a significant increase in its efficacy in eliciting anti-HSV-2-specific neutralizing antibody response and protective immunity against wild-type HSV-2 genital infection and disease in mice compared with a non-gD2-expressing non-replicating dominant-negative HSV-2 recombinant virus [13]. Moreover, compared with live CJ2-gD2, uv-inactivated CJ2-gD2 was ineffective in protecting against HSV-2 genital infection, indicating that de novo viral antigen expression by CJ2-gD2 is required for the demonstrated vaccine efficacy against HSV-2 infection [13].

Unlike the mouse model of HSV-2 genital infection in which spontaneous reactivation from latent infection rarely occurs in the vaginal tract, guinea pigs experience episodic spontaneous recurrent infection and disease following recovering from primary HSV-2 genital infection [14], [15]. Moreover, after primary HSV-2 intravaginal infection, guinea pigs develop vesicular lesions similar to those in humans, which include development, appearance, and duration of disease. In the current study, we investigated the vaccine efficacy of CJ2-gD2 in the guinea pig model of HSV-2 genital infection and whether CJ2-gD2 would represent a better vaccine candidate than the gD2 subunit vaccine formulated with alum/MPL in protecting against HSV-2 infection. We show that CJ2-gD2 is highly effective in preventing primary and recurrent genital herpes infections and disease in the immunized animals. Moreover, CJ2-gD2 is significantly superior to gD2-alum/MPL subunit vaccine in eliciting anti-HSV-2 specific neutralizing antibody response as well as protective immunity against primary and recurrent HSV-2 genital infections.

## Materials and Methods

### 2.1. Ethics Statement

All animal experiments conducted in this study were approved by the Harvard Medical Area Standing Committee on Animals and the American Veterinary Medical Association (Protocol Number: 03167), which is accredited by the Association for Assessment and Accreditation of Laboratory Animal Care (AAALAC) and meets National Institutes of Health standards as set forth in “The Guide for the Care and Use of Laboratory Animals.”

### 2.2. Cells, viruses and subunit vaccines

Vero cells were grown and maintained in Dulbecco's Modified Eagle's Medium (DMEM; Sigma) supplemented with 10% fetal bovine serum (FBS) [16]. U2OS cells are an human osteosarcoma line that can complement functionally for the HSV-1 and HSV-2 ICP0 deletions [13], [16]. U2CEP4R11 cells, an U2OS cells derived tetR-expressing cell line, were grown in DMEM containing 10% FBS and hygromycin B [17].

Wild-type HSV-2 strains, MS and 186, were propagated and plaque assayed in Vero cells. CJ2-gD2 is an HSV-2 strain 186-derived ICP0-null mutant-based non-replicating dominant-negative recombinant virus encoding the gD2 gene driven by the tetO-bearing HSV-1 ICP4 promoter and the UL9-C535C gene under the control of the truncated form of hCMVTO [13]. UL9-C535C consists of Met-Gly followed by the C-terminal amino acids 535-851 of the UL9 protein [18]. CJ2-gD2 was propagated and plaque assayed in U2CEP4R11 cells [13]. The titer of CJ2-gD2 stock used in this study was 1.45×10^8^ PFU/ml on U2CEP4R-11 cell monolayers.

The recombinant gD2 protein, produced from CHO cells stably expressing a truncated form of gD2 polypeptide consisting of amino acids 1 - 306 of the mature gD2 with NQG3H9 appended to the C-terminal, was purified at Merck Research Laboratories, West Point, PA [19]. The gD2 coding sequence is derived from HSV-2 strain G. The gD2-alum/MPL subunit vaccine was freshly prepared prior to each immunization in a formulation similar to that described by Bourne et al. [20] and Awashthi et al [19]. In brief, 30 µg of the recombinant gD2 protein was first mixed with 750 µg of alum (Imject Alum, Thermo Scientific, Rockford, IL) in a volume of 850 µl on a rotating platform. After 30 minuntes of incubation at room temperature, 75 µg of Monophosphoryl Lipid A (MPL) (Avanti Polar Lipids, Inc., Alabaster, AL) was added to gD2-alum solution followed by gentle mixing. MPL stock solution was prepared at a concentration of 500 µg/ml containing 10% DMSO (Sigma Aldrich) and store at -20°C (InvivoGen, San Diego, CA).

### 2.3. Immunization and challenge

Female Hartley guinea pigs (300-350 g) were obtained from Charles River Laboratories (Wilmington, MA). Animals were randomly assigned to three groups of eight animals each in the first experiment and three groups of 6six to eight each in the second experiment. Each of the groups was either sham-immunized with DMEM (n = 8, 8), immunized with gD2-alum/MPL at a dose of 3 µg of gD2/animal (n = 8, 6) as described by Hoshino et al. [21], or immunized with CJ2-gD2 at a dose of 5×10^6^ PFU/animal (n = 8, 6). Each vaccine was administered by i.m. into the quadriceps of the left and right hind limbs in a volume of 50 µl per injection [20], [22]. Consistent with the vaccination regimen in GSK's phase III clinical trials, in which gD2-alum/MPL subunit vaccine was administered 3 times [23], [24], guinea pigs were boosted with gD2-alum/MPL or CJ2-gD2 on days 14 and 28 post primary immunization. Anesthetized sham-immunized and immunized animals were preswabbed with a moist sterile calcium alginate swab (Calgiswab type 2, Puritan Medical Products Company LLC, Maine USA) and challenged intravaginally with 5×10^5^ PFU of HSV-2 strain MS at 3 weeks after the third immunization in experiment 1 and at 2 weeks after the third immunization in experiment 2 [22]. Since the demonstrated efficacy of each immunogen in protecting against HSV-2 genital infection and disease is essentially the same across both experiments, the results from the corresponding groups in each experiment have been combined and are presented together in this study.

### 2.4. Clinical observations

After challenge with wild-type HSV-2, the animals were examined daily until day 60 post-challenge. The number of lesions for individual animals was counted and the disease was scored non-blindly as previously described [22]: 0 =  no disease; 1 =  redness or swelling; 2 =  a few small vesicles; 3 =  several large vesicles; 4 =  several large ulcers with maceration; 5 =  paralysis; and 6 =  death.

### 2.5. Analysis of acute and recurrent vaginal shedding of challenge virus

Animals were anesthetized and vaginal mucosae were swabbed on days 1, 2, 3, 5, 7, and 9 post-challenge. Materials on individual swabs were suspended in 1 ml of DMEM containing 10% FBS in the presence of 100 U/ml penicillin G and 100 µg/ml of streptomycin sulfate (Gibco, Carlsbad, CA). Infectious virus on swab materials was assessed by standard plaque assay in 60-mm dishes of Vero cells. The minimum titer of challenge virus that could be detected was 1 PFU per original vaginal swab materials. For analysis of recurrent virus shedding, swabs were taken daily from days 21 to 50 post challenge. DNA was isolated from swab materials with the DNeasy tissue kit (Qiagen, Santa Clarita, CA), and stored at −20 C.

### 2.6. Neutralizing antibody assay

Blood was obtained from the saphenous veins at weeks 2, 4 and 6 or 7 post-primary immunization. HSV-2-specific neutralizing antibody titers in serum collected from each animal were determined in the presence of complement as described previously [20], [25]. In brief, serum prepared from individual animals was first heat-inactivated followed by a series of two-fold dilutions with normal growth medium containing amphotericin-B at 50 µg/ml and Low Tox M rabbit C (Accurate Chemical and Scientific, Westbury) at 15-fold final dilution. 250 PFU of wild-type HSV-2 strain 186 was added to the diluted serum or titration growth medium in the presence of Low Tox M rabbit C in a final volume of 600 µl. After incubation at 37°C for 1 h, HSV-2 titers were determined in duplicate 60-mm dishes of Vero cells by standard plaque assays under growth medium containing 5% FBS and 2% methylcellulose (M352-500, Fisher Scientific). The neutralizing titer was expressed as the final serum dilution required to achieve a 50% reduction in HSV-2 PFU relative to the HSV-2 PFU obtained in media plus complement alone. The results are presented as the mean ± SEM.

### 2.7. Western blot analysis

60-mm dishes of Vero cells in duplicate were mock-infected or infected with wild-type HSV-2 strain 186 at an MOI of 5 PFU/cell at 42 h post-seeding. Cell extracts were prepared at 16 h post-infection [16]. To further investigate immunization in eliciting gD-specific antibody response, U2OS cells were mock-transfected or transfected with a gD2-encoding plasmid, p02.4TO-gD2 [13] and cell extracts were prepared at 48 h post-transfection. Proteins in the cell extracts were resolved on SDS-PAGE followed by western blot analysis with guinea pig serum pooled from an equal volume of serum collected from sham control, CJ2-gD2-immunized animals (n = 8) or gD2-alum/MPL-immunized animals (n = 8) described in the first experiment in section 2.3 two weeks after the third immunization at a dilution of 1∶200. After 1 h incubation at room temperature, the membranes were washed and reacted with HRP-conjugated goat-anti-guinea pig IgG (sc-2438, Santa Cruz Biotechnology, Santa Cruz, CA). Similar results were obtained when the described western blot analyses were repeated or performed with pooled serums prepared from an equal volume of serum collected from CJ2-gD2-immunized animals (n = 6) or gD2-alum/MPL-immunized animals (n = 6) 2 weeks after the third immunization in the second experiment described under section 2.3.

### 2.8. Quantitative real-time PCR

On day 60 post-challenge, twelve lower lumbar and sacral dorsal root ganglia (DRG) per guinea pig were collected from individual guinea pigs immunized with CJ2-gD2 or gD2-Alum/MPL. The dorsal root ganglia DNA was extracted using the DNeasy tissue kit (Qiagen, Santa Clarita, CA), and suspended in 100 µl AE buffer. As a control, the DRG DNA was also isolated from four shamed-immunized guinea pigs euthanized on days 17, 18, 23, and 30, respectively, post-challenge due to the severity of local and systemic illness caused by HSV-2 infection. The presence of HSV-2 DNA was quantified by real-time PCR (Applied Biosystems 7300 Real-Time PCR System) with 250 ng of ganglia DNA or 50-100 ng vaginal swab DNA and primers specific to the HSV DNA polymerase (Forward: 5′ GCT CGA GTG CGA AAA AAC GTT C, Reverse: 5′ CGG GGC GCT CGG CTA AC) as previously described [12]. The minimal copies of HSV-2 viral DNA that could reliably be detected were one copy per reaction.

### 2.9. Statistical analysis

Un-paired Student's *t*-tests were performed in the statistical analysis of experimental results between sham-immunized animals and gD2-alum/MPL immunized animals, sham-immunized animals and CJ2-gD2-immunized animals, and gD2-alum/MPL-immunized animals and CJ2-gD2-immunized animals.

## Results

### 3.1. CJ2-gD2 is highly effective against primary and recurrent HSV-2 genital infections in guinea pigs

**Figure 1:**
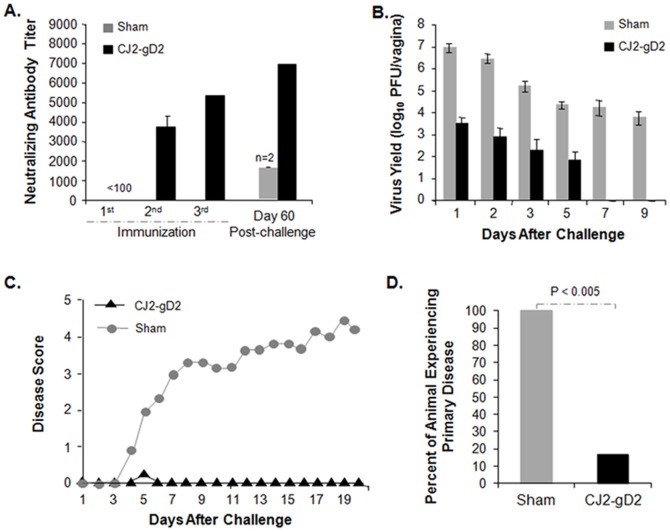
CJ2-gD2 is highly effective in eliciting HSV-2-specific neutralizing antibodies and protective immunity against HSV-2 primary infection and disease in guinea pigs. Female Hartley guinea pigs were randomly assigned to two groups of six animals each and were either sham-immunized with DMEM or immunized with CJ2-gD2 at a dose of 5×10^6^ PFU by intramuscular injection into the quadriceps of the left and right hind limbs on days 0, 15, and 29. A. Blood was taken on days 14, 28, and 49 after the primary immunization or 60 days after challenge and HSV-2-specific neutralizing antibodies in individual serums were determined. The results represent average titers ± SEM. B. Three weeks after the third immunization, guinea pigs were preswabbed and infected intravaginally with 5×10^5^ PFU of wild-type HSV-2 strain MS. Vaginal swabs were taken on days 1, 2, 3, 5, 7, and 9 post-infection. Virus yields in swab materials were determined by standard plaque assay on Vero cell monolayers and expressed as the mean ± SEM for individual vaginal swabs. C and D. Following infection with wild-type HSV-2, sham-immunized and CJ2-gD2-immunized guinea pigs were monitored daily from days 0 to 60 for the incidence of genital and disseminated HSV-2 disease. Presented is the disease score for the first 20 days after challenge (C) and the percent of animals that experienced primary herpetic disease (D).

As an initial study in evaluating the vaccine efficacy of CJ2-gD2 in protecting against HSV-2 genital infection and disease, female guinea pigs were first sham-immunized or immunized with CJ2-gD2 at a dose of 5×10^6^ PFU/animal three times at two-week intervals. At three weeks after the third immunization, the guinea pigs were preswabbed and challenged intravaginally with 5×10^5^ PFU of HSV-2 strain MS. The results in Fig. 1 show that immunization with CJ2-gD2 elicited a strong HSV-2-specific neutralizing antibody response after boost immunization (Fig. 1A). Importantly, the neutralizing antibody titers detected in CJ2-gD2-immunized animals were significantly higher than those of the two surviving sham-immunized animals at day 60 post-intravaginal challenge with wild-type HSV-2 (p<0.01). Immunization with CJ2-gD2 markedly reduced the acute intravaginal virus replication of the challenge virus in the immunized animals compared with the sham-immunized controls with a greater than 2,600-fold (p = 0.03) and 3,100-fold (p = 0.02) reduction in challenge virus yields on days 1 and 2 post-challenge, respectively (Fig. 1B). While all the sham-immunized controls continued to shed virus with an average yield of ∼15,800 PFU/ml on day 7 and ∼5,660 PFU/ml on day 9 post-challenge, no virus shedding was detected in 5 of 6 CJ2-gD2-immunized animals by day 3 post-challenge. The average duration of viral shedding in the CJ2-gD2-immunized guinea pigs was 2.5 days compared with greater than 9 days in the sham-immunized controls (p<0.001).

Consistent with these observations, CJ2-gD2 immunization is very effective in protecting against primary (Fig. 1C and 1D) as well as recurrent (Table 1) HSV-2 genital disease. While all the sham-immunized animals developed multiple genital herpes lesions following challenge with wild-type HSV-2 and four of six animals died by day 17 post-challenge, only 1 of 6 CJ2-gD2-immunized animals experienced a minor lesion on day 5, and that animal was recovered by the next day. Similarly, immunization with CJ2-gD2 led to a nearly 58-fold reduction in the incidence of recurrent disease by the challenge wild-type virus in the immunized animals compared with the sham-immunized controls between days 18 and 60 post-challenge (p = 0.001).

**Table 1:** Immunization with CJ2-gD2 is highly effective in protecting against recurrent HSV-2 genital disease and the establishment of latent infection.

		
	Recurrent disease (days 18–60)			Latent viral genome	
Group					
	Percent	Cumulative lesion/animal	Frequency a	Percent	Copy b
Sham (n = 2)	100%	19	9.5	100%	530, 27908
CJ2-gD2 (n = 6)	33%	0.33	0.33	0%	undetected

a Recurrent lesion days per animal between days 18 to 60 post challenge.

b Latent viral genome number per 250 ng DRG DNA in 2 surviving sham-immunized guinea pigs and CJ2-gD2-immunized animals on day 60 post challenge.

### 3.2. CJ2-gD2 is significantly superior to the gD2-alum/MPL subunit vaccine in eliciting HSV-2-specific neutralizing antibody response

**Figure 2:**
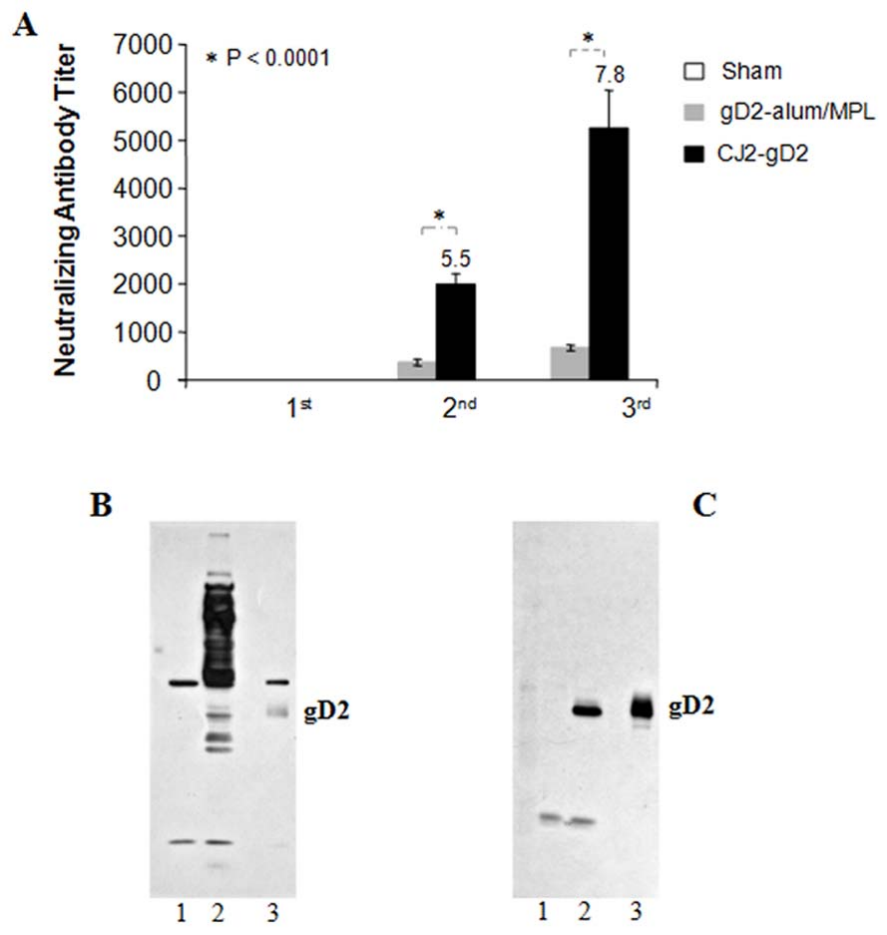
CJ2-gD2 is significantly more effective than gD2-alum/MPL subunit vaccine in eliciting an HSV-2-specific neutralizing antibody response in immunized guinea pigs. A. Female Hartley guinea pigs were randomly assigned to three groups of eight animals each in the first experiment and three groups of six to eight each in the second experiment. They were either sham-immunized with DMEM (n = 8, 8), immunized with 3 µg of purified recombinant gD2 freshly formulated with 7.5 µg of MPL and 75 µg of alum (n = 8, 6) or immunized with CJ2-gD2 (n = 8, 6) at a dose of 5×10^6^ PFU on days 0, 14, and 28. Blood was taken after the first, second, and third immunizations as detailed in the Materials and Methods. Heat inactivated serum from each animal was assayed individually for HSV-2-specific neutralizing antibody titers on Vero cell monolayers. The results represent average titers ± SEM. B and C. Western blot analysis with serum prepared from CJ2-gD2 (B)- and gD2-alum/MPL (C)-immunized guinea pigs as detailed in the Materials and Methods. Lanes 1, 2, and 3 represent cell extracts prepared from mock-infected Vero cells, HSV-2-infected Vero cells, and gD2-transfected U2OS cells, respectively.

Fig. 2A represents the combined results from two independent experiments, in which two sets of female Hartley guinea pigs were sham-immunized with DMEM (n = 8, 8), immunized with freshly prepared gD2-alum/MPL (n = 8, 6), or immunized with CJ2-gD2 (n = 8, 6). The neutralizing antibody titers detected in the CJ2-gD2-immunized animals are 5.5- and ∼8-fold higher than those detected in the gD2-alum/MPL-immunized animals after the second and third immunizations, respectively (p<0.0001). Again, there was a significant increase in the HSV-2 neutralizing antibody titers from the first to the second vaccination with an average titer of ∼1,990 after the second immunization and ∼5,260 after the third immunization. The average HSV-2 neutralizing antibody titer in gD2-alum/MPL-immunized animals was 364 after the second immunization and ∼670 after the third immunization. No HSV-2-specific neutralizing antibody was detected in serum from the sham-immunized animals at 1∶10-dilution.

The western blot analysis with pooled serum prepared from equal volume of gD2-alum/MPL immune animals (Fig. 2C) and CJ2-gD2 immune animals (Fig. 2B) described in the first experiment in section 2.3 demonstrates that in addition to the gD2-specific antibody response, immunization with CJ2-gD2 elicits strong antibody responses to various HSV-2 antigens. Expectedly, immunization with gD2-alum/MPL induced gD antibody response only. These results also reveal that although CJ2-gD2 is much less efficient in eliciting gD2-specific antibodies than gD2-alum/MPL, the overall HSV-2-antibody response elicited in CJ2-gD2-immunized animals was significantly higher than the monovalent gD2 antibody response induced in gD2-alum/MPL-immunized animals. Collectively, the results indicate that the high-level neutralizing antibody titer detected in CJ2-gD2-immunized animals can be attributed to its ability to elicit anti-gD2 antibody response as well as antibody responses to various other HSV-2 virion-associated envelop proteins. This finding is consistent with the observations of Hoshino et al. [26] and Halford et al. [27] reflecting that while immunization with gD2-alum/MPL induces potent gD-specific antibody response, gD2-alum/MPL is less superior in eliciting anti-HSV-2 neutralizing antibody response compared with HSV-2 recombinant virus-based vaccine candidates capable of eliciting antibody response to multiple HSV-2 surface antigens.

### 3.3. CJ2-gD2 is more effective than gD2-alum/MPL in protecting against acute replication of challenge HSV-2 as well as primary and recurrent HSV-2 genital disease

**Figure 3:**
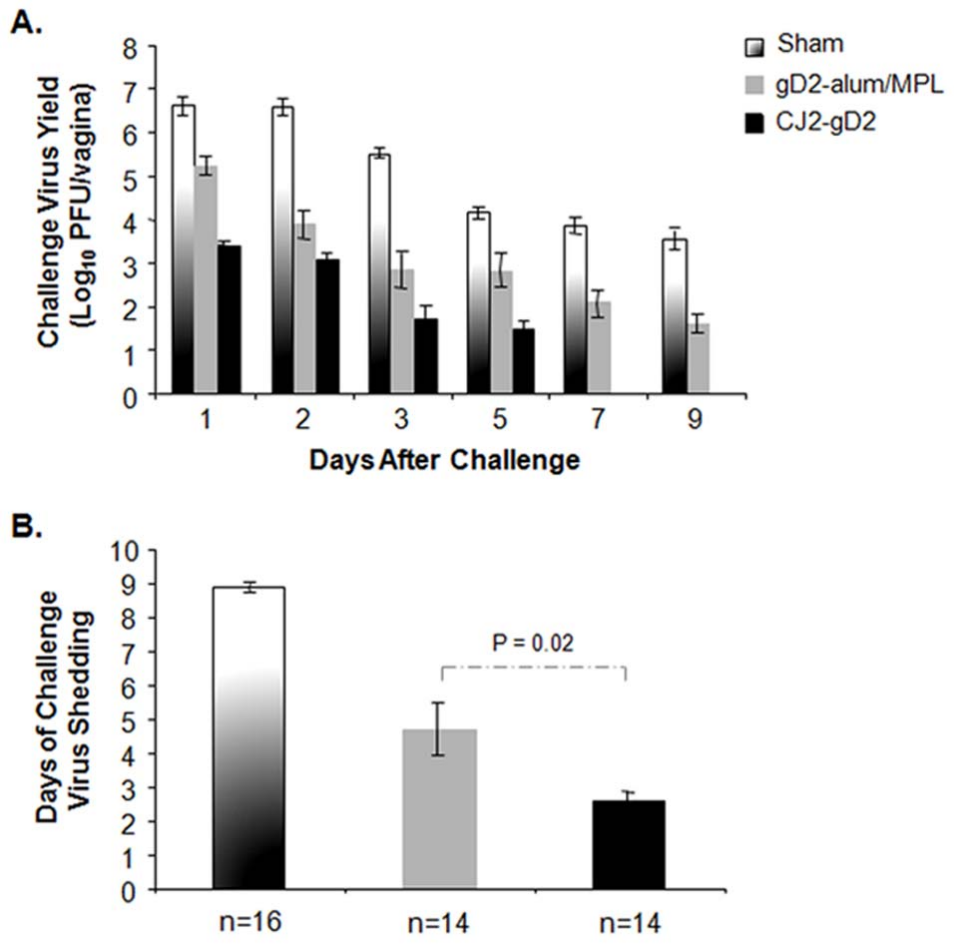
CJ2-gD2 exhibits superior vaccine efficacy than gD2-alum/MPL subunit vaccine in protecting against intravaginal wild-type HSV-2 infection in guinea pigs. Female guinea pigs sham-immunized with DMEM or immunized with gD2-alum/MPL or CJ2-gD2 described in Fig. 2 were challenged intravaginally with 5×10^5^ PFU of HSV-2 strain MS. Vaginal swabs were taken on days 1, 2, 3, 5, 7, and 9 post-challenge. Infectious virus on swab materials was determined by standard plaque assay in Vero cells. Viral yields are expressed as the means ± SEM for individual swabs (A). The duration of viral shedding is represented as the mean number of days during which infectious virus was detected in vaginal swabs ± SEM (B).

The sham-immunized and immunized animals described in Fig. 2A were challenged intravaginally with wild-type HSV-2 as detailed in the Materials and Methods. The results in Fig. 3A showed that the yields of challenge virus in sham-immunized controls were ∼4×10^6^ PFU/ml, ∼3.8×10^6^ PFU/ml, and ∼3.1×10^5^ PFU/ml on days 1, 2, and 3 post-challenge, respectively. The yields of challenge virus in the CJ2-gD2-immunized animals were 1,600- (p<0.0001), and 3,000-fold (p = 0.02) lower than the sham-immunized animals on days 1 and 2 post-challenge, respectively, and 71-fold lower than in the gD2-alum/MPL-immunized animals on day 1 post-challenge (p = 0.047). By day 5, 12 of 14 CJ2-gD2-immunized animals showed no virus shedding, while 50% of the gD2-alum/MPL-immunized animals continued to shed virus at an overall average yield of ∼650 PFU/ml. The duration of challenge virus shedding was 2.6 days in the CJ2-gD2-immunized animals compared with 4.7 days in the gD2-alum/MPL group (p = 0.02) and 8.9 days in the sham-immunized control (p<0.0001) (Fig. 3B).

**Figure 4:**
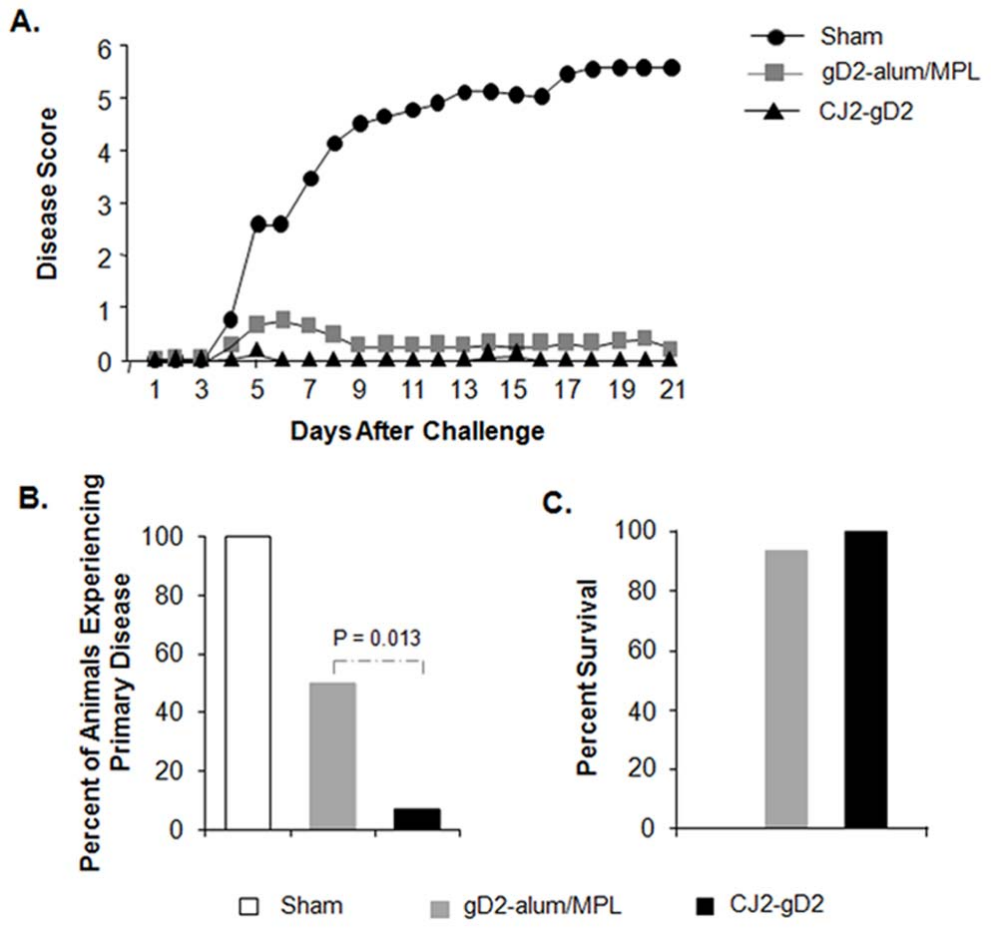
Prevention of primary HSV-2 genital disease in guinea pigs immunized with CJ2-gD2. Sham-immunized, gD2-alum/MPL- or CJ2-gD2-immunized guinea pigs described in Fig. 3 were monitored daily during a 60-day follow-up period for the incidence of genital and disseminated HSV-2 disease. The severity of disease was scored: 0, no sign of disease; 1, redness or swelling; 2, a few small vesicles; 3, several large vesicles; 4, several large ulcers with maceration; 5, paralysis; and 6, death. Presented are the disease scores for the first 21 days after challenge (A), the percent of animals that experienced primary herpetic disease (B), and the percent of survival until day 60 after challenge (C).

The impact of immunization with CJ2-gD2 and gD2-alum/MPL in protecting against primary skin lesions is summarized in Fig. 4. All the sham-immunized animals developed multiple genital herpetic lesions following challenge with wild-type HSV-2 and succumbed to HSV-2 infection by day 30 post-challenge. A total of 515 lesions were detected in the sham-immunized animals between days 5 and 7 post-challenge, while only one of fourteen CJ2-gD2 immune animals exhibited 1 mild herpetiform lesion on day 5 between days 1 and 11 post-challenge. Of the fourteen animals immunized with gD2-alum/MPL, seven experienced primary herpetic skin lesions with a total of 54 lesions detected between days 4 and 8. Moreover, one of the gD2-alum/MPL immunized animals had to be sacrificed on day 35 post-challenge due to the severity of local and systemic herpetic disease, whereas no CJ2-gD2-immunized animals showed any signs of neurological illness. Collectively, the results demonstrate that CJ2-gD2 is markedly superior to gD2-alum/MPL in protecting against HSV-2 primary genital disease (Fig. 4B, p = 0.013).

**Figure 5:**
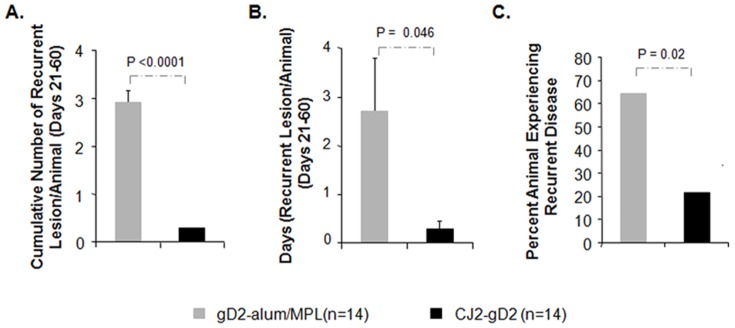
Prevention of recurrent HSV-2 disease in guinea pigs immunized with CJ2-gD2. After challenge with wild-type HSV-2, individual guinea pigs described in the legend of Fig. 4 were monitored daily during a 60-day follow-up period for the incidence of genital and disseminated HSV-2 disease. Presented are the cumulative numbers of recurrent lesions per animal (A), number of days that recurrent disease was experienced per animal (B), and the percent of animals that experienced recurrent disease between days 21 to 60 after challenge (C).

The results in Fig. 5 showed that immunization with CJ2-gD2 is significantly more effective than immunization with gD2-alum/MPL in protecting against recurrent HSV-2 genital disease in terms of cumulative recurrent lesions per animal (0.29 vs. 2.9, p<0.0001) and days on which animals exhibited recurrent disease (0.29 vs. 2.7, p = 0.046). In these studies, 79% of the CJ2-gD2-immunized animals experienced no detectable recurrent disease compared with 36% observed in the gD2-alum/MPL subunit vaccine group (p = 0.02).

Real-time PCR analysis of genital swab materials collected from days 21 to 50, however, revealed that immunization with CJ2-gD2 did not offer significantly better protection against recurrent virus shedding compared with immunization with the gD2 subunit vaccine. Specifically, ten of fourteen guinea pigs in the CJ2-gD2 immunized animals and nine of fourteen gD2-alum/MPL immunized animals showed no detectable recurrent virus shedding. The frequency of recurrent virus shedding from days 21 to 50 was 0.019 days/animal in the CJ2-gD2 group and 0.026 days/animal in the gD2-alum/MPL-immunized group (p = 0.67). The average number of HSV-2 genome detected in vaginal swabs per day from days 21 to 50 was 1.8 copies/animal in the CJ2-gD2 group and 3.2 copies/animal in the gD2-alum/MPL-immunized group (p = 0.25).

### 3.4. Immunization with CJ2-gD2 is highly effective in protecting against the establishment of latent infection by challenge HSV-2

**Figure 6:**
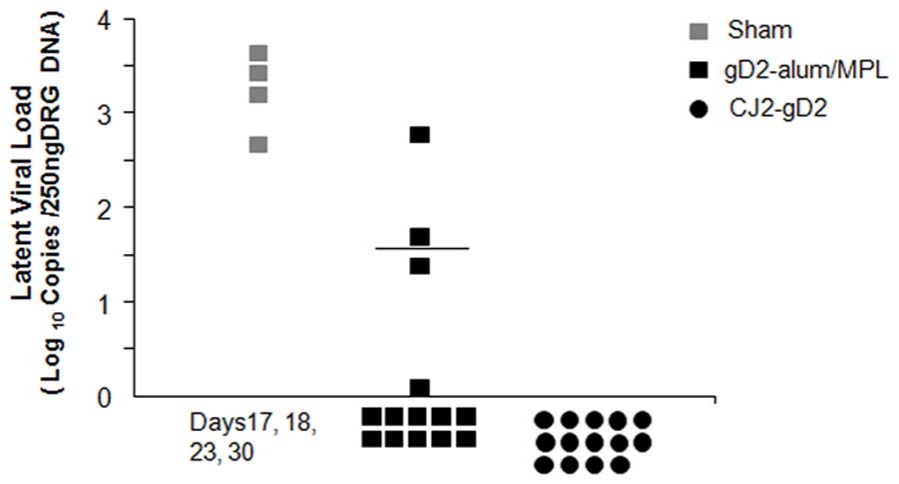
Protection from latent viral infection in guinea pigs immunized with CJ2-gD2. Sixty days after challenge with wild-type HSV-2, twelve lower lumbar and sacral dorsal root ganglia (DRG) per guinea pig were collected from all fourteen CJ2-gD2-immunized guinea pigs and the thirteen surviving gD2-alum/MPL-immunized guinea pigs. As a control, the DRG DNA was also isolated from four sham-immunized guinea pigs euthanized on days 17, 18, 23, and 30 post-challenge, respectively, due to the severity of local and systemic illness caused by HSV-2 infection. The total DNA was extracted and the presence of latent viral DNA was quantified by quantitative real-time PCR. The HSV-2 genome number per guinea pig was determined and presented as mean value. The guinea pig showing the highest viral DNA load in gD2-alum/MPL group was the one euthanized on day 35 post-challenge.

At day 60 post-challenge, the dorsal root ganglia DNA were harvested from all fourteen CJ2-gD2-immunized animals and the thirteen surviving gD2-alum/MPL immunized animals. The amount of viral DNA per guinea pig was determined by quantitative real-time PCR. No challenge HSV-2 viral DNA was detected in DRG DNA isolated from CJ2-gD2-immunized guinea pigs, while four of fourteen gD2-alum/MPL-immunized animals and all four sham-immunized animals euthanized on days 17, 18, 23, and 30 post-challenge, respectively, had detectable latent HSV-2 viral DNA (Fig. 6).

## Discussion

Various vaccine candidates have been investigated in the guinea pig model of HSV-2 genital infection [19], [28]–[30]. The subunit vaccine gD2/AS04, which contains recombinant gD2 in combination with the adjuvant alum and MPL, was effective in protecting against primary and recurrent HSV-2 genital disease in immunized animals following intravaginal infection with wild-type HSV-2 [20], [31]. While in earlier phase III clinical trials at an immunization dose of 20 µg of gD2 with 50 µg of MPL and 500 µg of alum, gD2/AS04 subunit vaccine provided 73–74% efficacy in protecting against genital herpes disease in HSV-seronegative women [23], no beneficial effect was observed in a recent phase III clinical trial [24]. Since gD2/AS04 elicits little or no CD8^+^ T-cell response [1], and because CD8^+^ T cells play a crucial role in controlling HSV infection [32]–[39], these studies have highlighted the importance of developing HSV vaccine candidates enabling the induction of broader and more robust humoral as well as CD4^+^ and CD8^+^ T-cell responses not only to gD2 but also to other HSV antigens [1], [7], [28], [40], [41].

Unlike the replication-defective HSV-2 recombinant vaccine constructs developed previously, i.e., *dl5-29* [42], which can co-replicate with wild-type virus or become replication competent in the context of wild-type HSV-1 and HSV-2 infection, CJ2-gD2 is a novel class of replication-defective HSV-2 recombinant viral vaccine that encodes a potent *trans*-inhibitory activity capable of blocking wild-type HSV-1 and HSV-2 viral replication in co-infected cells. In addition to its unique capability of expressing high levels of gD2 independent of HSV-2 viral DNA replication, CJ2-gD2 expresses a wide array of HSV-2 antigens, such as the viral immediate-early antigens ICP4, ICP27, ICP22; the early antigens UL23, UL29, UL39, UL50; and the early-late antigens UL18, UL19, UL49, UL55, and US8, which are the dominant targets of CD8^+^ T-cell response in HSV-2-infected individuals [43]–[45]. We show that immunization with CJ2-gD2 elicits significantly greater HSV-2-specific CD4^+^ and CD8^+^ T-cell responses in the immunized mice compared with the sham-immunized control animals after infection with wild-type HSV-2. Notably, the onset of the HSV-2-specific CD4^+^ T cell response in CJ2-gD2 immune mice is 2 days faster than the primary CD4^+^ T-cell response elicited in wild-type HSV-2 infected sham-immunized control [13].

HSV employs various strategies to evade host immune responses [46]–[53]. ICP0 and ICP47 are the two viral immediate-early proteins that involve in host immune evasion at a very early stage of viral infection. ICP0 plays a key role in blocking IFN-induced inhibition of viral infection [52], [53] as well as the TLR2/TLR9-mediated inflammatory cytokine response to HSV infection [54]. Thus, in comparison with HSV recombinant viral vaccines encoding functional ICP0, ICP0-null mutant-based vaccine candidates, such as CJ2-gD2, are likely more active in stimulating an innate host immune response, leading to enhanced vaccine efficacy against HSV infection [55]. We showed that immunization with CJ2-gD2 elicited strong antibody responses to various HSV-2 antigens and yielded HSV-2-specific neutralizing antibody titers 8-fold higher than those elicited by the gD2-alum/MPL subunit vaccine after three immunizations.

Hoshino et al. revealed that, compared with immunization with the gD2-alum/MPL, immunization with the replication-defective HSV-2 viral vaccine, *dl*5-29, offered slightly better efficacy in protecting against acute and recurrent HSV-2 genital disease in HSV-1-seronegative guinea pigs and *dl*5-29 was also more effective than the gD2-alum/MPL subunit vaccine in induction of HSV-2 neutralizing antibody response [21]. Notably, the same study showed that immunization with gD2-alum/MPL had little effect in protecting against acute challenge virus replication in the first 6 days post-challenge compared with sham immunization. Consistent with findings of Bourne et al. [20], [31] and Awashthi et al. [19], in which animals were immunized with gD2-alum/MPL twice or three times at a dose of 5 µg of gD2, we showed that immunization with gD2-alum/MPL was very effective in protecting against HSV-2 intravaginal infection. Compared with sham immunization, immunization with the gD2-alum/MPL led to 23-fold (p = 0.0001), 508-fold (p = 0.019), 442-fold (p = 0.07), and 23-fold (p = 0.0001) reduction in challenge virus replication on days 1, 2, 3, and 5 post-challenge, respectively. Significantly, immunization with CJ2-gD2 is more robust in blocking acute challenge virus replication than the gD2-alum/MPL subunit vaccine. Yields of challenge virus in CJ2-gD2-immunized animals were ∼70-fold lower than gD2-alum/MPL-immunized animals on day 1 post-challenge, and 1600- and 3000-fold lower than sham-immunized control on days 1 and 2 post-challenge, respectively. The duration of challenge virus shedding was reduced to 2.6 days in CJ2-gD2-immunized animals compared with 4.7 days in gD2-alum/MPL group and 8.9 days in sham-immunized animals.

In accordance with its superiority in eliciting protective immunity against acute HSV-2 replication, immunization with CJ2-gD2 is also markedly superior to the gD2-alum/MPL in protecting against primary as well as recurrent HSV-2 genital disease. First, immunization with CJ2-gD2 provided nearly 100% protection against primary genital lesions after challenge with wild-type HSV-2. While 50% of gD2-alum/MPL-immunized animals experienced primary herpetic skin lesions with a total of 54 lesions detected between days 4 and 8, only one of the fourteen CJ2-gD2 immune animals exhibited a single mild herpetiform lesion from days 1 to 11 post-challenge. Second, CJ2-gD2 immunization resulted in significantly lower rates of recurrent disease in the immunized animals than did gD2-alum/MPL immunization in terms of the incidence, cumulative recurrent lesions per animal and days in which animals exhibited recurrent disease. Third, while recurrent disease could be observed in gD2-alum/MPL vaccinated animals as late as day 50 post-challenge, no recurrent disease and recurrent virus shedding were detectable after day 37 post-challenge in CJ2-gD2-immunized animals. Lastly, no latent HSV-2 viral DNA was detectable in DRG of CJ2-gD2-immunized animals harvested on day 60 after challenge (Table 1 and Fig. 6); in contrast, four of fourteen gD2-alum/MPL immunized animals had detectable latent HSV-2 viral DNA.

It has been documented that both *dl*5-29 and the gD2-alum/MPL subunit vaccine are effective in reduction of recurrent HSV-2 genital disease in HSV-1-seropositive guinea pigs [21]. Moreover, *dl*5-29 has been shown effective as a therapeutic vaccine in reducing recurrent HSV-2 genital disease in guinea pigs previously infected by HSV-2 [26]. Given that like *dl5-29*, CJ2-gD2 is a replication-defective virus incapable of de novo viral DNA replication, and its unique ability to express high levels of gD2 independent of viral DNA replication, it is reasonable to expect that CJ2-gD2 could also be an effective vaccine in protecting against HSV-2 genital infection in HSV-1 seropositive guinea pigs as well as a therapeutic vaccine against recurrent HSV-2 genital infection and disease in guinea pigs.

To date, no vaccine capable of completely preventing HSV infection has been reported. The induction of both effective mucosal and systemic immune responses is likely required for optimal protection against HSV genital infection. Given the recent studies that intranasal immunization of gD/liposome complex [56] or gD-IgG2a Fc fusion protein in CpG [57] can effectively elicit HSV-2-specific mucosal immunity, it would be of great interest to test whether the efficacy of CJ2-gD2 in eliciting mucosal immune response can be enhanced by a prime/boost regimen consisting of CJ2-gD2 and gD2 subunit vaccine [58], [59].
